# Genetic Control of Susceptibility to Infection with *Candida albicans* in Mice

**DOI:** 10.1371/journal.pone.0018957

**Published:** 2011-04-20

**Authors:** Irena Radovanovic, Alaka Mullick, Philippe Gros

**Affiliations:** 1 Biochemistry Department, McGill University, Montréal, Québec, Canada; 2 Biotechnology Research Institute, Montréal, Québec, Canada; Louisiana State University, United States of America

## Abstract

*Candida albicans* is an opportunistic pathogen that causes acute disseminated infections in immunocompromised hosts, representing an important cause of morbidity and mortality in these patients. To study the genetic control of susceptibility to disseminated *C. albicans* in mice, we phenotyped a group of 23 phylogenetically distant inbred strains for susceptibility to infection as measured by extent of fungal replication in the kidney 48 hours following infection. Susceptibility was strongly associated with the loss-of-function mutant complement component 5 (*C5*/*Hc*) allele, which is known to be inherited by approximately 40% of inbred strains. Our survey identified 2 discordant strains, AKR/J (C5-deficient, resistant) and SM/J (C5-sufficient, susceptible), suggesting that additional genetic effects may control response to systemic candidiasis in these strains. Haplotype association mapping in the 23 strains using high density SNP maps revealed several putative loci regulating the extent of *C. albicans* replication, amongst which the most significant were C5 (P value = 2.43×10^−11^) and a novel effect on distal chromosome 11 (P value = 7.63×10^−9^). Compared to other C5-deficient strains, infected AKR/J strain displays a reduced fungal burden in the brain, heart and kidney, and increased survival, concomitant with uniquely high levels of serum IFNγ. C5-independent genetic effects were further investigated by linkage analysis in an [A/JxAKR/J]F2 cross (n = 158) where the mutant *Hc* allele is fixed. These studies identified a chromosome 11 locus (*Carg4*, *Candida albicans* resistance gene 4; LOD = 4.59), and a chromosome 8 locus (*Carg3*; LOD = 3.95), both initially detected by haplotype association mapping. Alleles at both loci were inherited in a co-dominant manner. Our results verify the important effect of C5-deficiency in inbred mouse strains, and further identify two novel loci, *Carg3* and *Carg4*, which regulate resistance to *C. albicans* infection in a C5-independent manner.

## Introduction

*Candida albicans* is an opportunistic fungal pathogen that exists commensally in the gastrointestinal and genitourinary tracts of healthy individuals[1], but that causes severe disseminated and often lethal infections in immunocompromised patients, such as those suffering from HIV infection or undergoing cancer chemotherapy[2]. In the United States alone, *Candida* species constitute the fourth most common causative agent of nosocomial bloodstream infections, and are associated with significant attributable mortality in both adults and children (47% vs. 29%)[3]–[5]. Despite the rising trend of infections with non-*albicans Candida* species, *Candida albicans* remains the most common isolate recovered from bloodstream infections worldwide, with the frequency of occurrence ranging from 37% to 70%[6]. Genetic effects have long been suspected to play a role in the initial susceptibility and subsequent development of severe *C. albicans* infection in humans[7] and in animal models of experimental infections[8], [9]. Genetic predisposition to disseminated candidiasis in non-immunocompromised humans has not yet been associated to any particular gene, although individuals presenting impaired phagocyte function are more susceptible to *Candida* infections[10], as observed in myeloperoxidase (MPO) deficiency. In addition, deleterious mutations in multiple direct or downstream immune effectors, notably CLEC7A[11], STAT3[12], and CARD9[13], have been found in human cohorts with high prevalence of chronic mucocutaneous candidiasis (CMC) and have been recapitulated and studied in mice.

In mouse models of infection, response to *C. albicans* is under a complex genetic control that affects onset of infection, type and severity of disease developed and associated pathologies (oropharyngeal, mucosal, or systemic forms), and extent of immune response elicited[14]. Inbred mouse strains vary widely in their degree of innate susceptibility to systemic candidiasis, being either highly susceptible (A/J, DBA/2) or highly resistant (BALB/c, C57BL/6J). Studies in inbred strains[15], [16], together with genetic linkage and association studies in informative backcross and F2 mice, and experiments in AcB/BcA recombinant congenic strains[17] derived from susceptible A/J and resistant C57BL/6J progenitors, have identified a critical role for the complement component 5 (C5) in differential susceptibility of these two inbred mouse strains. A/J and other susceptible strains carry a defective *C5* allele, which causes susceptibility to infection with *C. albicans*, as well as *Cryptococcus neoformans* [18], [19]. Phenotypically, resistant C5-sufficient C57BL/6J mice die late in infection due to high kidney fungal loads, and associated strong neutrophil-driven inflammatory response at that site, while C5-deficient A/J mice succumb within 24 hrs of infection with little kidney damage, but displaying an allergic-like response associated with high levels of circulating TNFα, IL-6, MCP-1, MCP-5 and eotaxin[20], [21], resulting in multiple organ failure including cardiomyopathy[21]. The complement pathway represents the first line of defense of the innate immune system and plays a major role in eliciting an inflammatory response to the site of infection[22]–[24]. The complement system can be activated by several pathways triggered by microbial products, which ultimately result in the activation of C3 convertase, cleavage of C5, release of chemotactic factors (C3a and C5a), and generation of the membrane attack complex (MAC)[23]. The importance of complement cascade is further demonstrated in C3^−/−^ animals which show impaired fungal clearance and higher mortality than wild type controls when infected with *C. albicans* or *C. glabrata* [25].

Studies in inbred strains and in AcB/BcA recombinant congenic strains have suggested that the deleterious effect of the *C5* allele on fungal load and survival time of *C. albicans* infected mice may be further modulated by genetic background effects[9], [15], [17]. In addition, the genetic analysis of histopathological responses in target organs following systemic *C. albicans* infection has pointed to C5-unrelated genetic loci, temporarily given the appellation *Carg1* and *Carg2* [26], [27]. Although the genes underlying these effects remain unknown, these studies have clearly pointed at additional complexity in the genetic control of host response to *C. albicans* infection. With the aim of identifying such additional gene effects, we have herein phenotyped a total of 23 phylogenetically distant inbred strains of mice for susceptibility to *C. albicans*. These studies have identified two strains (AKR/J and SM/J) that show discordant genotype/phenotype relationships with respect to C5 status and susceptibility to infection. Haplotype association mapping in the 23 inbred strains using high density SNP maps, together with genetic linkage analyses in informative crosses derived from discordant strains, have uncovered two C5-independent genetic effects and loci controlling fungal replication in the host, and mapping to chromosomes 8 (*Carg3*), and 11 (*Carg4*).

## Results

### Response to *C. albicans* infection in inbred mouse strains

**Figure 1:**
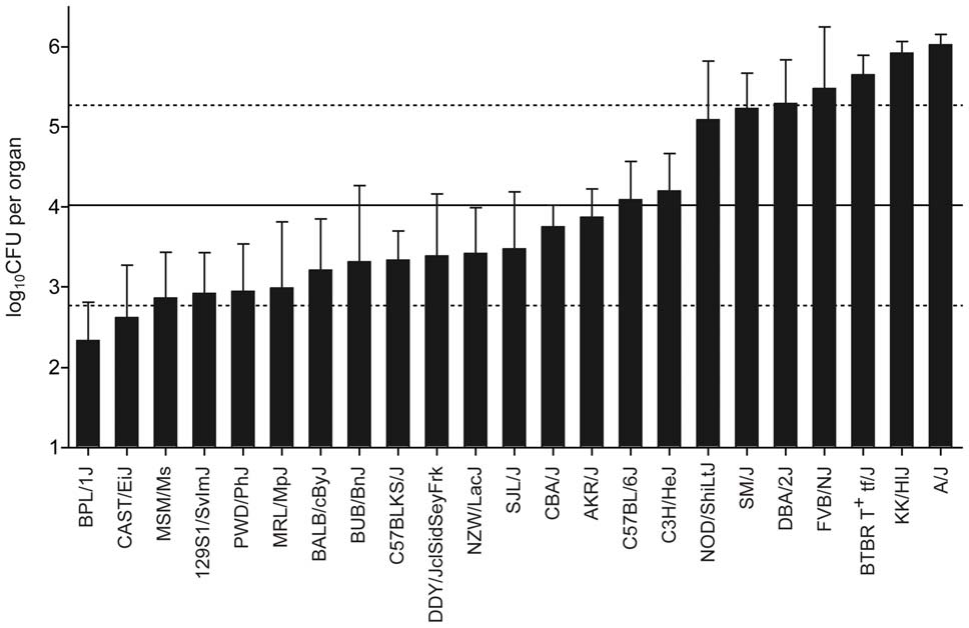
Kidney fungal burden, a measure of susceptibility to *C. albicans* infection, in 23 inbred mouse strains. 23 out of 36 commonly used inbred strains from the Jackson Mouse Phenome Database were phenotyped for susceptibility to *C. albicans*. 7–10 female mice per strain were infected intravenously with 5×10^4^ *C. albicans* blastospores and the kidney fungal load was measured 48 h post-infection. Bars represent strain mean±SD. Horizontal lines represent mean (solid line) across all 23 strains±SD (dashed lines).

To identify novel C5-independent genetic effects regulating the proliferation of *C. albicans* organisms in target organs during disseminated infection, we surveyed 23 strains from the panel of 36 commonly used inbred mouse strains represented in the Jackson Mouse Phenome Database. These strains have been selected to be genetically diverse, on the basis of their phylogenetically distinct breeding background, and thus likely to be representative of the natural allelic pool. The mice were challenged intravenously with a low dose of *C. albicans* SC5314 and the fungal replication was assessed in the kidney 48 h following infection. We observed wide differences in the extent of *C. albicans* colonization and replication in kidneys of these inbred strains (Figure 1), ranging from very low levels (log_10_CFU = 2.3±0.5) such as in the BPL/1J strain, to approximately 10 000-fold greater number of fungi in the highly susceptible A/J (log_10_CFU = 6.0±0.1), along with a number of strains showing a wide range of intermediate phenotypes. To determine the impact of the C5 locus and its mutant *Hc* allele on the response to *C. albicans* infection in these strains, we established the C5 genotype of the 23 strains (Table 1). We also segregated strains into a susceptible group (log_10_CFU>5.1) consisting of 7 strains and a resistant group (log_10_CFU<4.2) consisting of 16 strains. Using this arbitrary segregation, we noted a very strong effect and near perfect correlation of C5 allelic status and extent of replication of *C. albicans* in kidney with two notable exceptions (Table 1). Despite being C5-sufficient and producing serum C5a during infection (data not shown), SM/J mice display a significantly higher mean fungal load (log_10_CFU = 5.2±0.4) when compared to the reference B6 strain (log_10_CFU = 4.1±0.5) or other C5-sufficient strains. AKR/J mice are C5-deficient [28], yet they showed mean log_10_CFU counts at 3.9±0.4 similar to that seen in the resistant B6 strain, and clearly distinct from that seen in the C5-deficient strains group (log_10_CFU>5.1).

**Table 1:** Phenotypic response of inbred mouse strains to *C. albicans* infection, with respect to the C5 status.

Inbred strain	Hc allele^a^	Number of mice (females)	Log kidney CFU (mean ± s. d.)	Phenotype^b^
A/J	0	9	6.0±0.1	S
KK/HlJ	0	10	5.9±0.2	S
BTBR T+ tf/J	0	10	5.7±0.3	S
FVB/NJ	0	10	5.5±0.8	S
DBA/2J	0	10	5.3±0.5	S
SM/J*	1	7	5.2±0.4	S
NOD/ShiLtJ	0	10	5.1±0.7	S
C3H/HeJ	1	10	4.2±0.5	R
C57BL/6J	1	8	4.1±0.5	R
AKR/J*	0	10	3.9±0.4	R
CBA/J	1	8	3.8±0.3	R
BALB/cByJ	1	7	3.5±0.6	R
SJL/J	1	8	3.5±0.3	R
NZW/LacJ	1	10	3.4±0.6	R
DDY/JclSidSeyFrkJ	1	8	3.4±0.8	R
C57BLKS/J	1	8	3.4±0.3	R
BUB/BnJ	1	8	3.3±1.0	R
MRL/MpJ	1	8	3.0±0.8	R
129S1/SvImJ	1	10	2.9±0.5	R
PWD/PhJ	1	9	2.9±0.6	R
MSM/Ms	1	8	2.9±0.6	R
CAST/EiJ	1	10	2.6±0.6	R
BPL/1J	1	8	2.3±0.5	R

Inbred mouse strains are classified by the extent of the kidney fungal burden and susceptibility (S) or resistance (R). AKR/J and SM/J mouse strains, denoted by *, were found to be discordant. All strains were genotyped to confirm their C5 allele.

a 0-C5 deficient; 1-C5 sufficient.

b R-resistant; S-susceptible.

*Discordant strains.

Although these results confirm the critical impact of the *C5* mutant allele on the response of inbred strains to disseminated *C. albicans* infection, the wide variation of CFU counts observed within mouse strains bearing wild type *C5* alleles and the presence of two clearly discordant strains (AKR/J, SM/J), together strongly suggested the presence of C5-independent genetic effects in the cohort of 23 strains tested.

### Chromosomes 2 and 11 are highly associated with response to *C. albicans* infection in inbred mouse strains

**Figure 2:**
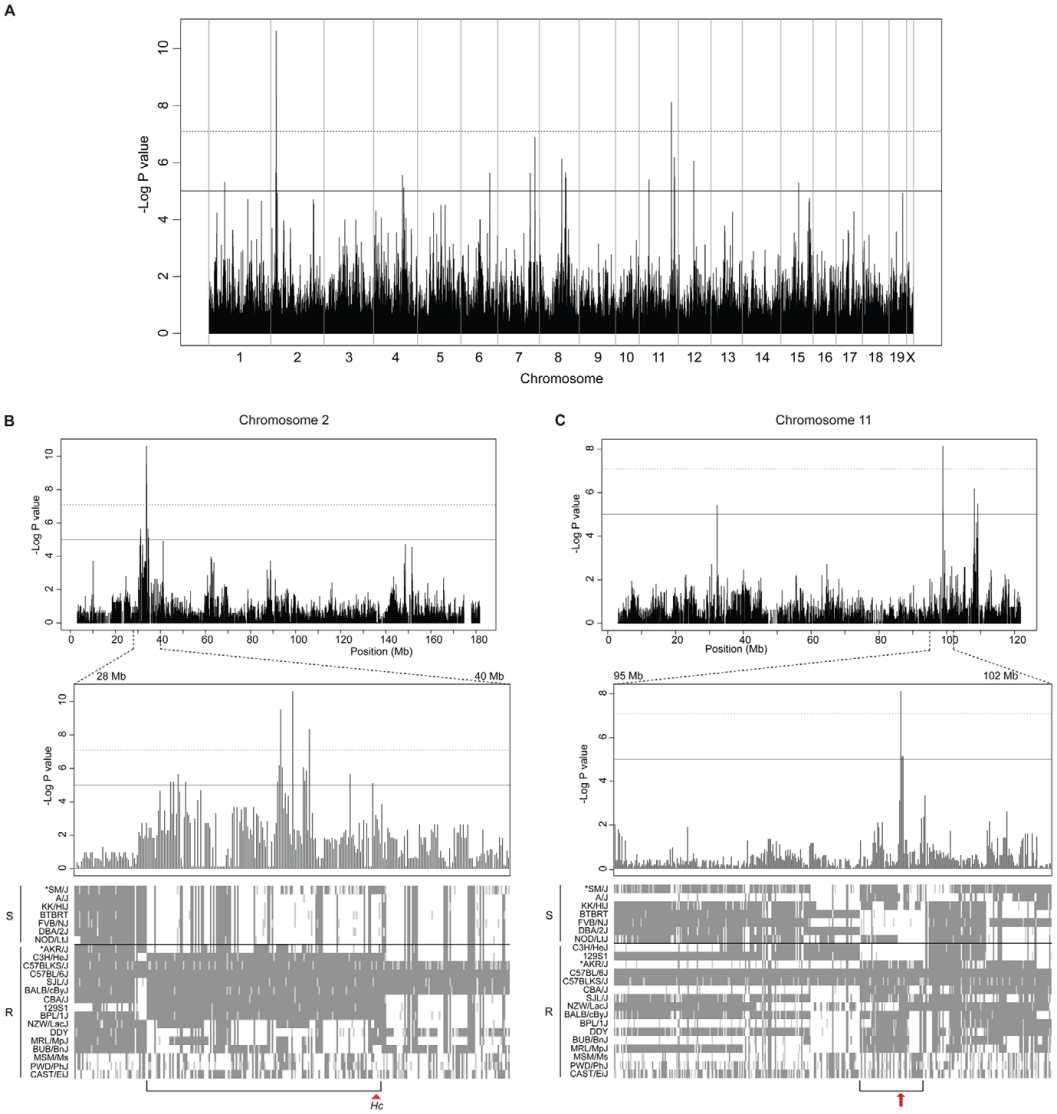
Genome-wide association mapping using EMMA. Using the R package implementation of EMMA, genome-wide association mapping was conducted across 23 inbred mouse strains. We obtained genotype data consisting of 132 000 SNPs from the Mouse HapMap project and rejected SNPs with MAF <0.05, which yielded a final set of 125 113 SNPs. These were then correlated with the kidney fungal burden as the phenotypic data and consequently, the analysis yielded −log_10_ transformed P values indicating association significance for each SNP genome-wide (A). Standardized threshold was set at P value 1.0×10^−5^ (solid line) and the Bonferroni multiple testing correction threshold was calculated to be 7.99×10^−8^ (dashed line). The most significant hits on chromosomes 2 and 11 are depicted (B and C) along with respective haplotype maps of susceptible (S) and resistant (R) strains at those locations. The conserved haplotype blocks are indicated with brackets, along with the *Hc* position (B) and the arrow showing the most conserved area (C). Asterisk denotes discordant strains with respect to the C5 genotype. Haplotype map colour coding: light gray (NA), gray (A/J like), and white (B6 like).

To explore the genetic basis for inter-strain differences in response to *C. albicans*, we used the strain distribution pattern (kidney CFU counts), and high density SNP datasets available for the 23 strains (Mouse HapMap SNP data) to perform whole genome association mapping. We utilized a statistical method EMMA [29] that corrects for population structure and genetic relatedness between inbred strains by estimating a kinship matrix. EMMA analysis identified multiple significant loci (Figure 2A, P value < 1×10^−5^) on chromosomes 1, 2, 4, 6, 7, 8, 11, 12, and 15 (Table S1). The Bonferroni correction [30] was used to provide a very conservative threshold (α = 0.01, P value = 7.99×10^−8^) which identified SNPs on chromosomes 2 and 11 as the most highly associated with CFU counts. As expected, the top 3 significant SNPs (P value =  2.43×10^−11^, 3.04×10^−10^, 4.65×10^−9^) are situated between 33.74 and 33.96 Mb on chromosome 2, and correspond to the *C5* gene located less than 1 Mb away. Repeating the EMMA analysis after removing phenotypically discordant AKR/J and SM/J strains, increases the significance of the Chr. 2 (C5) locus dramatically (P value = 6.60×10^−19^, Figure S1). Examination of the haplotype structure of chromosome 2 near the peaks of highest association (Figure 2B) shows segregation of the A/J-type allele (30.26–34.88 Mb) in all susceptible strains, except for SM/J. Conversely, the AKR/J strain carries the A/J haplotype block, but is resistant. The structure of the Chr. 2 haplotype block is more difficult to follow in wild-derived strains MSM/Ms (*M. m. molossinus*), PWD/PhJ (*M. m. musculus*), and the CAST/EiJ (*Mus castaneus*). The next most significant association (P value = 7.63×10^−9^) was detected on the distal portion of chromosome 11 (98.87 Mb). Haplotype analysis of the corresponding Chr. 11 shows a haplotype block less conserved in inbred strains than the Chr. 2 haplotype, including multiple possible recombination events within the haplotype map (Figure 2C). Nevertheless, a small haplotype block (97.84-99.44 Mb) is preserved where most of the resistant strains, including the AKR/J strain, carry B6 alleles. These results imply that although C5 plays a crucial role in response to *C. albicans* infection, several additional loci, including the locus on chromosome 11, can modulate response to infection in inbred strains.

### Phenotypic expression of resistance to *C. albicans* infection in AKR/J mice

**Figure 3:**
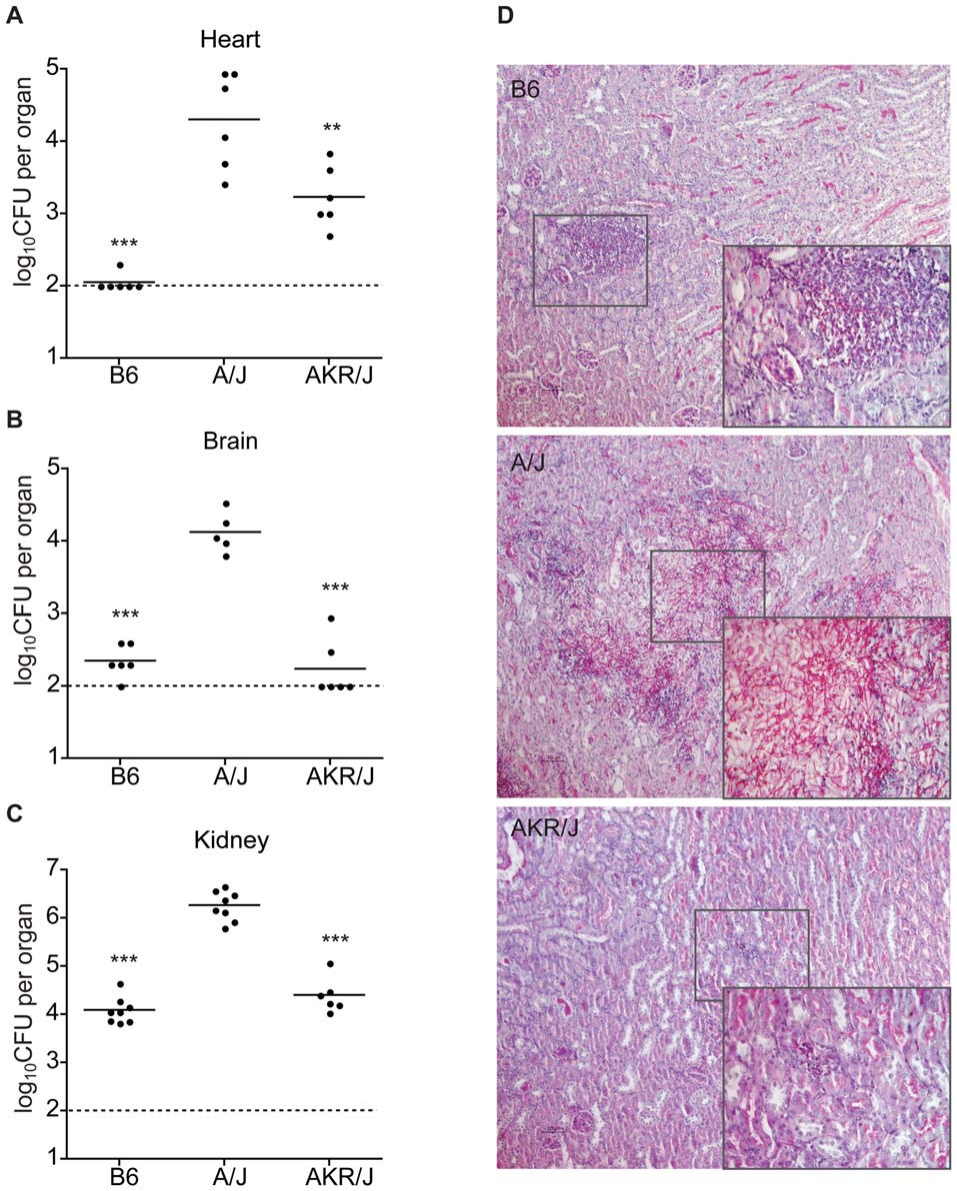
Phenotypic responses of discordant AKR/J mouse strain upon *C. albicans* infection. In 6 mice per strain, the heart (A), brain (B), and kidney (C) fungal load was measured 48 h after a low dose (5×10^4^, i.v.) infection with *C. albicans*. In all organs, fungal load was significantly lower in AKR/J mice compared to A/J (**P<0.01, ***P<0.0001). Periodic acid-Schiff staining was performed on kidney sections (D) showing the extent of fungal burden and granulocyte infiltration. Magnification 100X and 400X (insert).

The differential susceptibility of A/J and AKR/J mouse strains to *C. albicans* infection occurs despite relatively close phylogeny and genetic relatedness of the two strains as assessed by the fraction of concordant alleles based on 8.27 million SNPs distributed across the genome [31]. Frazer *et al.* [31] have reported this fraction to be 0.899 between A/J and AKR/J strains. To explore further the phenotypic expression of AKR/J-associated resistance, C57BL/6J, A/J and AKR/J mice were challenged intravenously with 5×10^4^ *C. albicans* blastospores, and fungal replication was assessed 48 h post-infection in brain, heart and kidney. Compared to A/J susceptible controls, AKR/J mice showed a significantly lower fungal load in brain (log_10_CFU: 4.1±0.3 vs. 2.2±0.4; Figure 3B) and kidney (log_10_CFU: 6.3±0.3 vs. 4.4±0.4; Figure 3C). Histological analysis of kidney sections (Figure 3D) showed massive proliferation of the *C. albicans* hyphae and moderate recruitment of cellular infiltrates in A/J mice. Kidney sections from B6 and AKR/J mice also showed cellular infiltrates and presence of pseudohyphal fungal elements, but to a much lesser extent than observed in A/J. Although the heart fungal burden was significantly reduced in AKR/J mice compared to A/J mice (log_10_CFU: 3.2±0.4 vs. 4.3±0.7; Figure 3A), it was nevertheless significantly higher than that of B6 (log_10_CFU: 2.1±0.1). We have previously established that C5-deficiency is associated in A/J mice with a striking cardiac phenotype, prior to and during *C. albicans* infection [21]. Therefore, it is possible that the intermediate level of *C. albicans* replication may be linked to such a heart-specific effect of C5-deficiency present in AKR/J. Together, these results strongly suggest that AKR/J mice are able to contain *C. albicans* replication in all organs, despite C5-deficiency.

**Figure 4:**
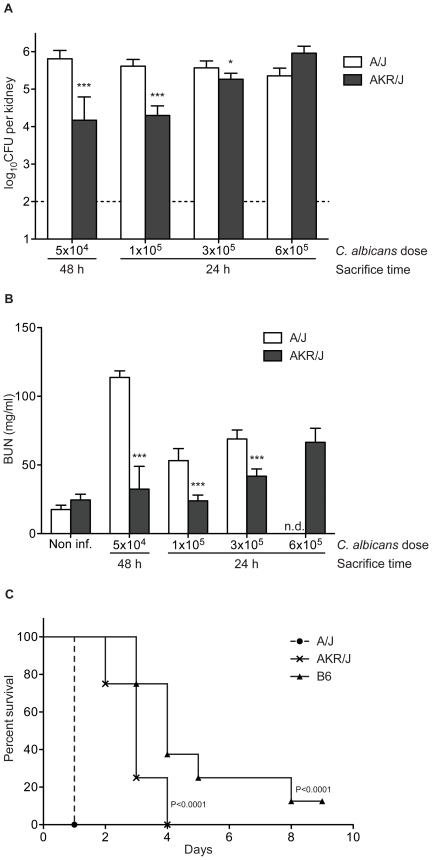
Differential susceptibility of A/J and AKR/J mice to *C. albicans* infection. 6 mice per strain were challenged with the indicated dose of *C. albicans* and sacrificed at either 24 h or 48 h time point to determine the kidney fungal burden (A) and serum BUN levels (B). The fungal load was significantly lower in AKR/J mice compared to A/J, and was associated with lower BUN levels (*P<0.05, ***P≤0.0001, n.d. not done due to premature death of A/J mice). Bars represent group mean ± SD. For the survival study, 8 mice per strain were infected intravenously with a high dose (3×10^5^) of *C. albicans* and monitored daily for clinical signs of morbidity (C). Survival curves were compared by a Log-rank test and the median survival of AKR/J (3 d) was found to be significantly different from A/J (1 d) (P<0.0001).

We further challenged AKR/J and A/J mice with several infectious doses of *C. albicans*, (5×10^4^, 1×10^5^, 3×10^5^, and 6×10^5^ blastospores) and monitored in these animals: a) organ CFUs, b) kidney function as measured by BUN (blood urea nitrogen), and c) survival (Figure 4). With respect to fungal load, the inter-strain difference between AKR/J (resistant) and A/J (susceptible) is clearly visible and is dose-dependent; it is most visible at lower doses and fades at the highly infectious dose. Moreover, these observations are paralleled by measurements of kidney function: BUN levels were always lower in the AKR/J strain (retained kidney function), and this for all infectious doses tested. Finally, we challenged A/J, AKR/J, and B6 mice with a high dose of *C. albicans* (3×10^5^, i.v.) as done previously and monitored survival daily. All A/J mice succumbed after 24 hours (highly susceptible), whereas the median survival time (MST) for AKR/J mice was 3 days (significant by a Log-rank test; p<0.0001), while MST for resistant B6 mice was 4 days.

**Figure 5:**
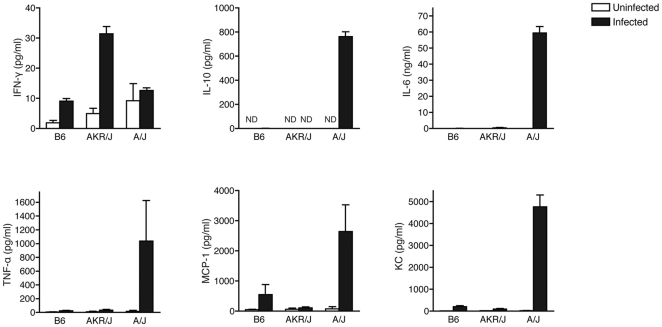
Inflammatory cytokine levels in the AKR/J mouse strain. Serum cytokine levels in 3–6 mice of each strain were measured prior to infection and at the 48 h time point, as described in Materials and methods. Cytokine response of AKR/J mice resembled closely that of B6 strain and was significantly different from A/J mice. The standard deviation is indicated for each group. For all cytokines, infected AKR/J shows a statistically significant difference from infected A/J mice.

Pro-inflammatory cytokines IL-6, TNF-α, and MCP-1 are found at high concentrations in circulating blood and kidney of C5-deficient mice (exemplified by A/J) at 24 and 48 h following *C. albicans* infection [20], [32], and elevated KC levels in kidney correlate with subsequent damage to this organ [32]. We compared serum cytokine profiles of A/J, AKR/J and C57BL/6J prior to and 48 h following *C. albicans* infection using the BD™ CBA Flex system (Figure 5). As expected, we observed high serum levels of pro-inflammatory cytokines IL-6, TNF- α, MCP-1, and KC as well as the Th2-specific cytokine IL-10 in infected A/J mice [33], [34]. On the other hand, and similar to B6 animals, AKR/J mice had undetectable levels of IL-10 even 48 h post-infection and no statistically significant increase in concentrations of IL-6, TNF-α, MCP-1 or KC. Interestingly, we observed a strong induction and elevated serum levels of IFNγ in infected AKR/J mice, which were significantly higher than those measured in infected C57BL/6J (resistant) and A/J (susceptible) controls. Studies in IFNγ mutant mice have previously demonstrated a protective effect of this Th1 cytokine against disseminated candidiasis [35], suggesting that the elevated levels of this cytokine during infection may have a protective effect in C5-deficient AKR/J mice.

### Genetic analysis of the *C. albicans* resistance trait of AKR/J mice

**Figure 6:**
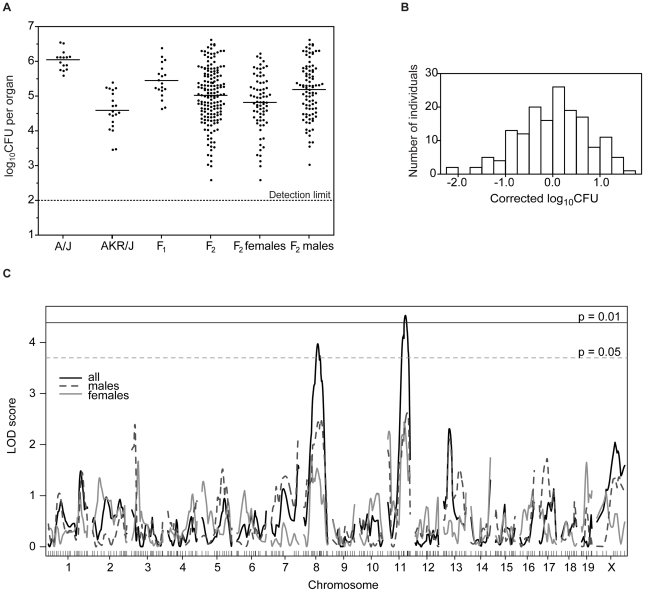
Linkage analysis in the informative [A/JxAKR/J]F2 population. [A/JxAKR/J]F2 mice (n = 158) were infected with *C. albicans* (5×10^4^, i.v.) and kidneys were harvested 48 h post infection. CFU were determined as previously mentioned and results from four separate infections are plotted along with A/J, AKR/J, and [A/JxAKR/J]F1 controls (A). Each dot represents a single mouse. Distribution of kidney fungal load is shown in the entire [A/JxAKR/J]F2 population (B), after regression of log_10_CFU to an experiment and gender-specific mean (set at 0). Mice were genotyped at 257 SNPs and markers across the entire genome and interval mapping was carried out using the R/qtl software package. Whole genome LOD score traces are shown for genetic effects controlling kidney fungal burden in [A/JxAKR/J]F2 mice (C), identifying linkage to chromosome 8 (LOD = 3.95) and chromosome 11(LOD = 4.59), designated *Carg3* and *Carg4*, respectively. Results for males and females are shown separately (dashed and dotted lines) and together (solid line), with marker positions on the x-axis. Permutation testing (1000 tests) identified genome-wide thresholds at P = 0.01 and 0.05.

**Figure 7:**
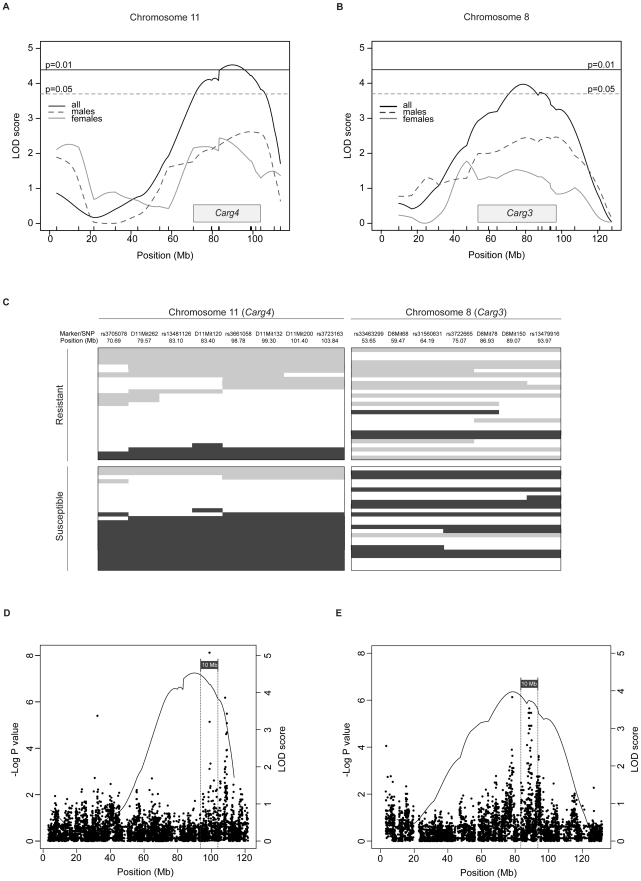
Additive effect of the *Carg3* and *Carg4* loci on kidney fungal burden in [A/JxAKR/J]F2 mice. Detailed LOD score traces are shown for chromosome 11 (*Carg4*) (A) and chromosome 8 (*Carg4*) (B) loci for males, females, and both sexes combined. The shaded area designates the Bayesian 95% confidence interval for each locus and the genome-wide thresholds are indicated at P = 0.01 and P = 0.05. The additive effect of *Carg3* and *Carg4* loci is demonstrated by segregating the alleles of susceptible and resistant mice found at extremities (±1 standard deviation from the mean) of the distribution (C). Haplotype map colour coding: light gray (AKR/J), gray (A/J), and white (heterozygous). Each line represents a single mouse. Genome-wide association mapping results are depicted along with LOD score traces for chromosomes 11 (D) and 8 (E). The 10 Mb blocks examined for candidate genes are indicated for both loci.

The inheritance and complexity of the C5-independent genetic control to *C. albicans* resistance of AKR/J mice was investigated by phenotyping mice from an informative F2 cross generated between resistant AKR/J and susceptible A/J progenitors, and in which all mice are fixed for C5 deficiency. [A/JxAKR/J]F1 hybrids showed an intermediate level of susceptibility with respect to kidney fungal load (Figure 6A) compared to the parental controls, suggesting that resistance to *C. albicans* in AKR/J mice is inherited in a co-dominant fashion. A total of 158 [A/JxAKR/J]F2 animals were infected intravenously with 5×10^4^ *C. albicans* blastospores in four separate infections (Figure 6A). The kidney fungal burden was determined and used as a quantitative phenotypic measure of susceptibility in linkage analyses. The results of four infections were combined and regressed in an e×periment- and sex-dependent manner, where the mean was set at 0. The distribution frequency (Figure 6B) and the log_10_CFU counts of [A/JxAKR/J]F2 mice (Figure 6A) show a normal distribution, with an approximate 1:1 ratio of resistant to susceptible mice confirming a co-dominant pattern of inheritance. Informative F2 mice were genotyped with a custom panel of 257 polymorphic informative markers (SNPs and microsatellites) distributed across the genome. Whole-genome multiple regression linkage analysis in R/qtl was performed for males (N = 96) and females (N = 72), or both genders together (Figure 6C). This analysis identified a highly significant linkage associated with fungal burden in the kidney on chromosome 11 (LOD = 4.59, P<0.01) and chromosome 8 (LOD = 3.95, P<0.05) (Figure 7A and 7B). These loci contributing resistance to *C. albicans* in the AKR/J strain were given a temporary identification *Carg3* (*Candida albicans* resistance gene 3) for chromosome 8 QTL and *Carg4*, for chromosome 11 QTL. The Bayesian 95% credible intervals were determined to be 53.6–96.9 Mb for *Carg3* and 70.7–103.8 Mb for *Carg4*, whereas the peak LOD scores were identified at 75 Mb (peak SNP: rs3722665) and 98.8 Mb (peak SNP: rs3661058), respectively. Both *Carg3* and *Carg4* loci were present amongst the initial SNP associations uncovered by EMMA analysis (Figure 2A and Table S1) and were found to be significant (P value<1×10^−5^), however, only *Carg4* passed the stringent Bonferroni correction. These results confirm results of the haplotype mapping studied in the 23 inbred strains, with respect to the effect of the *Carg4* locus on response to disseminated *C. albicans* infection. They also suggest that the *Carg4* locus strongly contributes to the noted resistance of AKR/J mice, acting independently of the C5 locus, for which susceptibility alleles are fixed in the analyzed AKR/JxA/J cross.

To examine whether these loci acted in an additive or epistatic manner, we performed a two-dimensional Haley-Knott multiple regression analysis using R/qtl. This analysis revealed that the joint effect of *Carg3* and *Carg4* increased the LOD score to 11.5, suggesting an additive effect which explains 28% of phenotypic variance observed. This additive effect is clearly illustrated when the extremities of the F2 distribution are segregated according to the more significant *Carg4* locus (Figure 7C) and exhibit a predominance of the AKR/J and A/J alleles in the resistant and susceptible F2 mice, respectively. Indeed, mice carrying the A/J allele at both *Carg3* and *Carg4* loci display a significantly higher kidney fungal load than mice carrying AKR/J alleles at both loci (P<0.0008, t-test). Although a few select resistant F2 animals carry the A/J allele at one locus, they consistently bear a non-A/J allele (AKR/J or heterozygous) at the other locus. The opposite is also true for susceptible F2 mice which inherited the AKR/J allele at either *Carg3* or *Carg4*. Finally, to illustrate the overlap achieved by EMMA and linkage analysis, association scores for chromosome 11 and 8 are depicted along with respective LOD score traces (Figure 7D and 7E). These results point to *Carg3* and *Carg4* as a novel two-locus system regulating C5-independent resistance to *C. albicans* infection in AKR/J mice.

## Discussion

We have used a representative panel of 23 phylogenetically distant inbred mouse strains to study the genetic control of susceptibility to acute and disseminated *C. albicans* infection. At the time of starting this survey, the critical impact of the mutant *C5* deficiency allele, fixed in certain inbred strains, on susceptibility to infection has been firmly established [17]. Additional genetic effects, in the form of the unmapped *Carg1* and *Carg2* loci, had suggested that additional genetic effects may further modulate response of inbred strains to this infection [26], [27], [36]. Our inbred strains survey produce two key findings. Firstly, it readily identified two strains that show discordant genotype/phenotype relationships with respect to C5 status and susceptibility to infection (Figure 1 and Table 1), namely AKR/J which carries the mutant *C5* allele but is resistant to infection, and SM/J which is susceptible despite a wild type allele at *C5*. Secondly, haplotype association mapping in the 23 inbred strains using kidney fungal load as a quantitative measure of susceptibility uncovered several candidate loci (in addition to C5 on Chr. 2) controlling fungal replication in the host. Subsequent genome scan in informative F2 mice generated between susceptible A/J and resistant AKR/J (fixed for mutant *C5* alleles) progenitors identified two highly significant linkages on chromosomes 8 (Carg3, LOD score = 3.95, P value<0.05) and 11 (*Carg4*, LOD score = 4.59, P value<0.01), as regulating permissiveness to *C. albicans* in these mice. *Carg3* and *Carg4* are novel loci that regulate susceptibility to *C. albicans* in a C5-independent fashion.

Genome-wide haplotype association in inbred strains of mice has proven to be a useful experimental strategy to detect loci that regulate complex traits [37], [38]. The limitations of this type of analysis reside in the relatively small number of inbred strains that were surveyed[29], [39], which limits the power of association study using single marker mapping (SMM) strategy [40]. SMM assigns a bi-allelic genotype to individual SNPs, and can therefore incorrectly model SNPs that possess three or even four predominant genotypes in inbred strains. This limits both the power of detecting relevant genetic effects, and the accuracy with which the corresponding loci and associated haplotype can be delineated. Also, loci that do not explain a substantial percentage of phenotypic variance[29] may go unnoticed. Therefore, the results of our analysis probably represent an underestimate of the number of genetic effects regulating susceptibility to *C. albicans* in inbred strains. A noteworthy advantage of the EMMA algorithm is the avoidance of inflation of false positives due to a kinship matrix that corrects for genetic relatedness amongst inbred strains[29]. Su *et al.* [39] and other groups[41] have acclaimed the strength of combining genome-wide association mapping and linkage analysis in an F2 cross to identify QTLs underlying complex traits and avoid false-positive loci. We created an informative [A/JxAKR/J]F2 cross in order to corroborate the loci identified by EMMA analysis, but in the context of C5 deficiency. Linkage mapping in the [A/JxAKR/J]F2 progeny identified a novel gene effect located on chromosome 8 and termed *Carg3* (LOD = 3.95) and confirmed the chromosome 11 locus (*Carg4*, LOD = 4.59) (Figure 6). In fact, the peak associated SNPs on chromosomes 8 and 11 are located directly below the significant LOD score traces and overlap with *Carg3* and *Carg4*, respectively (Figure 7C, 7D) strongly suggesting that the same loci were detected by both approaches.

Recently, it was demonstrated that elevated levels of IL-6, MIP-1β, and mostly KC in kidney extracts of *C. albicans* infected mice correlate with kidney damage [32]. This is exemplified by the susceptible A/J strain (Figure 5), which displays an acute and possibly pathological inflammatory response upon systemic *C. albicans.* The phenotypic response to infection in C5-deficient AKR/J mice with respect to serum cytokine profiles is clearly distinct from that of C5-deficient A/J mice, but rather resembles that of resistant and C5-sufficient C57BL/6J strain, with one notable exception. Indeed, infected AKR/J mice show strikingly elevated levels of serum IFNγ compared to either resistant C57BL/6J or susceptible A/J. Although we cannot yet establish that elevated serum IFNγ seen in AKR/J are solely or partly responsible for resistance in this strain, a protective role for this cytokine in *C. albicans* infection has been established. Indeed, mice deficient for IFNγ[35] or IFNγ receptor[42] show increased susceptibility to *C. albicans*, and passive administration of IFNγ[43] causes a reduction in tissue fungal burden in normal mice. In addition, *in vitro* studies have also shown that IFNγ potentiates phagocytosis and killing of *C. albicans* by neutrophils[44] and macrophages[45]. Moreover, IFNγ is used as a prophylactic treatment of infection in patients with chronic granulomatous disease (CGD), a genetically inherited disease characterized by an increased susceptibility to fungal infections[46]. Therefore, we hypothesized that elevated levels of this cytokine during infection may have a protective effect in C5-deficient AKR/J mice by directly potentiating the fungicidal effect of immune cells and/or stimulating host genes implicated in antifungal defense and which underlie *Carg3* and *Carg4* loci.

Signaling by IFNγ activates the JAK-STAT pathway, which leads to the binding of phosphorylated STAT1 to IFNγ activation site (GAS) elements and also interferon-stimulated response elements (ISREs)[47], [48]. Robertson *et al.* recently used chromatin immunoprecipitation and DNA sequencing to map all functional STAT1 binding sites (and associated genes) which are induced by IFNγ treatment of human HeLa S3 cells [49]. We therefore investigated the *Carg3* and *Carg4* for the presence of positional candidates that may be bound by STAT1 in response to IFNγ. The minimal genetic interval inferred by linkage analysis at *Carg3* extends over 40 Mb, while that at *Carg4* is 30 Mb. As both regions are extremely gene rich, we restricted the size of the genomic candidate regions by combining linkage analysis and the haplotype association mapping results. Therefore, we prioritized for analysis blocks of 10Mb centered around the SNPs showing the highest scores in the haplotype association studies for both loci (Figure 7D). This analysis identified a total of 59 (*Carg3*) and 147 such genes (*Carg4*) (Tables S2 and S3). We also superimposed on this dataset, those genes whose mRNA expression has been shown by transcript profiling to be modulated at 2h, 4h, or 6h following exposure to IFNγ [50]. An intersection of genes containing high STAT1 binding ChIP-Seq peaks (identified by combination of chromatin immunoprecipitation and DNA sequencing; ChIP/seq) and genes whose mRNAs were significantly modulated by IFNγ, yielded a list of 4 and 11 genes. We consider these genes as priority candidate genes for the *Carg3* and *Carg4* effects.

Four genes in the *Carg3* interval fulfill both criteria: *Adcy7*, *Dnaja2*, *Gab1* and *Inpp4b*. *Adcy7* is of particular interest for the following reasons. *Adcy7* codes for an isoform of adenylate cyclase that is expressed at high levels and regulates the intracellular levels of cyclic adenosine monophosphate (cAMP) in macrophages, T cells and B cells [51], [52]. cAMP is as a potent immunosuppressor in antigen-presenting cells and T lymphocytes acting as an intracellular signal to dampen synthesis of pro-inflammatory cytokines in these cells [53]-[55]. For example, cAMP acts as a second messenger for the anti-inflammatory action of prostaglandin E2 (PGE2) in macrophages, which ultimately leads to reduced phagocytosis, decreased production of reactive oxygen species, attenuation of TNFα, MIP-1α, LTB4 secretion, and increased IL-10 production by these cells. *Adcy7* expression is abundant in macrophages, stimulated by IFNγ, and associated with STAT-1 binding to the *Adcy7* gene promoter (Table S2). Tissue-specific ablation of *Adcy7* in the hematopoietic system causes a defect in cAMP production, and is associated with increased production of TNFα by macrophages following exposure to LPS *ex vivo*, and hyper-sensitivity to LPS-induced endotoxin shock *in vivo* [51]. Therefore, it is tempting to speculate that high levels of circulating IFNγ in AKR/J mice may stimulate expression of *Adcy7* in immune cells, with concomitant effects on intracellular cAMP levels and intensity of inflammatory response in these mice. The noted absence of infiltrating inflammatory cells in the kidneys of *C. albicans* infected AKR/J mice (Figure 3D) is consistent with a dampened inflammatory response associated with elevated adenylate cyclase activity and cAMP levels [56]. Additional experimentation will be required to formally test this proposal.

Amongst the list of 11 *Carg4* positional candidates whose expression is regulated by IFNγ and which display IFNγ regulated STAT1 recruitment to their promoters (Table S3), we note the presence of *Ifi35*. Ifi35 belongs to the family of interferon-inducible proteins, a group of proteins whose expression is rapidly induced by both type I and type II interferons, and that have been associated with different pathologies including systemic lupus erythematosus [57], cancer [58], and antiviral host defense [59]. IFI35 is a leucine zipper-containing transcriptional regulator which is normally found in the cytoplasm of cells, and is translocated to the nucleus following exposure to IFNγ[60]. In the nucleus, IFI35 heterodimerizes with other proteins (Nmi, CKIP-1)[61], including the transcription factor B-ATF[62]. The physical interaction between B-ATF (basic leucine zipper transcription factor, ATF-like) and IFI35 suggests a role for IFI35 in cytokine signaling, and early polarization of the T helper response[62]. B-ATF is a member of the AP-1 family of transcription factors expressed primarily in hematopoietic cells[62], [63]. B-ATF binds to the promoter of the *Il17* gene and plays a critical role in Th17 differentiation of helper T cells[63]. Indeed, *Batf^-/-^* deficient mice display normal Th1, and Th2 response but are deficient in Th17 differentiation, and are resistant to experimental autoimmune encephalitis[63]. Il17 and Th17 differentiation of T cells is essential for protection against fungal pathogens, and Il17^-/-^ mice are hyper-susceptible to infection with *C. albicans* [64]. In addition, alterations in Th17 response have been associated with susceptibility to candidiasis in human clinical cases[13], [65], [66]. Therefore, Ifi35 may play a role in anti-fungal defenses by modulating Th17 response, a hypothesis that can be tested experimentally.

In conclusion, we have combined genome-wide association mapping and linkage analysis to identify and validate two novel loci that modulate response to systemic *C. albicans* infection in mice. These two loci exert their effect in a complement C5-independent fashion. The identification of the genes involved should provide valuable insight into host defenses against acute candidiasis.

## Materials and Methods

### Mice

Inbred strains 129S1/SvlmJ, A/J, AKR/J, BALB/cByJ, BPL/1J, BTBRT+tf/J, BUB/BnJ, C3H/HeJ, C57BL/6J, C57BLKS/J, CAST/EiJ, CBA/J, DBA/2J, DDY/JclSidSeyFrkJ, FVB/J, KK/HlJ, MRL/MpJ, MSM/Ms, NOD/ShiLtJ, NZW/LacJ, PWD/PhJ, SM/J, and SJL/J were obtained as pathogen-free mice at 8–12 weeks of age from the Jackson Laboratory (Bar Harbor, ME) as part of a collaboration with the Mouse Phenome Database project. Survey data will be deposited in MPD (www.phenome.jax.org) and made publically available. [A/JxAKR/J] F2 progeny were bred by systematic brother-sister mating of [A/JxAKR/J] F1 mice. All housing and experimental procedures were conducted under the guidelines of the Canadian Council of Animal Care and were approved by the Biotechnology Research Institute (BRI) Animal Care Committee (Protocol number: 08-SEP-I-017) and the Animal Care Committee of McGill University (Protocol number: 5618).

### Infection with Candida albicans

*C. albicans* strain *SC5314* was grown overnight in YPD medium (1% yeast extract, 2% Bacto Peptone and 2% dextrose) at 30°C. Blastospores were harvested by centrifugation, washed twice in phosphate-buffered saline (PBS), counted using a hemacytometer and resuspended in PBS at the required density. For experimental infections, mice were inoculated via the tail vain with 200 µl of a suspension containing 5×10^4^ *C. albicans* blastospores in PBS. In dose-response experiments, additional doses were tested: 5×10^4^, 1×10^5^, 3×10^5^, and 6×10^5^ *C. albicans*. Forty-eight hours following infection (unless otherwise indicated), target organs were removed aseptically and homogenized in 5 ml of PBS. The homogenate was then serially diluted and plated on YPD-agar plates containing 34 µg/ml of chloramphenicol. The plates were incubated at 30°C for 24–48 hours and the colony-forming units (CFU) counted and expressed as log_10_CFU per organ. The maximum sensitivity for this assay was 100 CFU, and the animals displaying titers below the detection limit were assigned an arbitrary value of 100 CFU.

For the survival study, mice were injected intravenously with 200 µl of a suspension containing 3×10^5^ *C. albicans* blastospores in PBS. Mice were closely monitored for clinical signs such as lethargy, hunched back, and ruffled fur. Mice exhibiting extreme lethargy were deemed moribund and were euthanized.

### Cytokine detection

Mice were anesthetized and exsanguinated by cardiac puncture. Serum was isolated by collection of blood, followed by centrifugation and storage at -80°C until used to measure cytokine levels. To determine the levels of cytokines in the circulation, 12.5 µl of serum was analyzed using the BD™ CBA Flex sets according to the manufacturer’s instructions. Fluorescence levels were recorded using the BD™ LSRII flow cytometry system (Becton-Dickinson Biosciences, CA, USA) using BD FACSDiva acquisition software and the data analysis was carried out using the FCAP Array software.

### Evaluation of kidney function

At 24 h or 48 h, mice were exsanguinated by cardiac puncture under anesthesia, and blood was collected in microtubes with separation gel (Sarstedt, Montreal, Canada). Serum was isolated by centrifugation and stored at −20°C until used for blood urea nitrogen (BUN) determination. A commercially available kit that allows quantitative urease/Berthelot determination was used to measure BUN levels (Sigma).

### Histology

Whole kidneys were fixed in 10% neutral buffered formalin, dehydrated in ethanol/xylene and embedded in paraffin, as described previously [67]. Histological sections were cut longitudinally at 5 µm on a microtome and fixed onto glass slides. Sections were deparaffinized, then stained with periodic acid Schiff’s reagent (PAS) to detect *C. albicans* elements and counterstained with hematoxylin to visualize immune cells infiltration. Stained sections were examined under a light microscope at 100X and 400X magnification and photographed.

### Genotyping

As described previously[68], genomic DNA was isolated from tail clips of individual F2 mice collected at the time of sacrifice. A total of 158 [A/JxAKR/J]F2 mice were genotyped at the McGill University and Genome Quebec Innovation Centre (Montreal, QC, Canada) using Sequenome iPlex Gold technology and a custom panel that contained 225 informative SNPs distributed across the genome. Additional microsatellite markers were obtained from the Mouse Genome Informatics Database (www.informatics.jax.org) and used for gap filling and fine mapping by a standard PCR-based method employing (α-^32^P) dATP labeling and separation on denaturing 6% polyacrylamide gels.

### Linkage analysis

QTL mapping was performed using Haley-Knott multiple regression analysis[69] or EM maximum likelihood algorithm[70]. A two-dimensional scan was performed using the two-QTL model and empirical genome-wide significance was calculated by permutation testing (1000 tests). All linkage analysis was performed using R/qtl[71].

### EMMA scan

The detailed algorithm underlying the efficient mixed-model for association mapping have been previously published[29]. The EMMA algorithm is based on the mixed-model association where a kinship matrix accounting for genetic relatedness between inbred mouse strains is estimated and then fitted to the vector of the phenotype, thereby reducing false positive rates. Prior to the analysis, a minor allele frequency cutoff (MAF<0.05) was applied. In order to identify the most highly associated loci, the Bonferroni multiple testing correction[30] was computed. EMMA is publically available as an R package implementation.

### Statistical analysis

An unpaired, two-tailed Student’s t-test was used to establish significance of differences in mean CFU per organ, BUN levels, and cytokine concentrations between different mouse strains. Survival of AKR/J mice was analyzed by a Log-rank test and survival fractions were compared using the χ^2^ statistic. These data were analyzed using GraphPad Prism 4.0 statistical software. P-values<0.05 were considered significant.

## Supporting Information

Figure S1 **Genome-wide association mapping without discordant AKR/J and SM/J strains.** EMMA analysis was conducted as described earlier (see Materials and Methods, and Figure 2) while omitting the AKR/J and SM/J discordant strains. -Log_10_P values are depicted and represent genome wide significance of association for each SNP. Standardized threshold was set at P value 1.0×10^−5^ (solid line) and the Bonferroni multiple testing correction threshold was calculated to be 7.87×10^−8^ (dashed line).
(PDF)

Table S1 ***In silico*** **identified loci controlling response to** ***C. albicans*** **infection in inbred mouse strains.** SNPs that have passed the Bonferroni cutoff (α = 0.01, P value  =  7.99×10^−8^) are shaded in gray.
(PDF)

Table S2 **Candidate genes in the** ***Carg3*** **region.** A total of 59 genes containing IFNγ-inducible STAT1 binding sites and their overall mRNA expression (>2X) upon IFNγ stimulation in Hela cells are represented. Genes considered for further prioritization had a high (>20) ChIP-Seq peak height and a significant (>2X) gene expression. N/A designation in the gene expression column was given for genes that were not represented on the microarray.
(PDF)

Table S3 **Candidate genes in the** ***Carg4*** **region.** A total of 147 genes containing IFNγ-inducible STAT1 binding sites and their overall mRNA expression (>2X) upon IFNγ stimulation in Hela cells are represented. Genes considered for further prioritization had both high (>20) ChIP-Seq peak height and a significant (>2X) gene expression. N/A designation in the gene expression column was given for genes that were not represented on the microarray.
(PDF)
