# Supplementary figures and images for: Genetic Control of Susceptibility to Infection with Candida albicans in Mice
Source: PLoS One. 2011 Apr 20;6(4):e18957. doi: 10.1371/journal.pone.0018957 (PMC3080400; doi:10.1371/journal.pone.0018957)

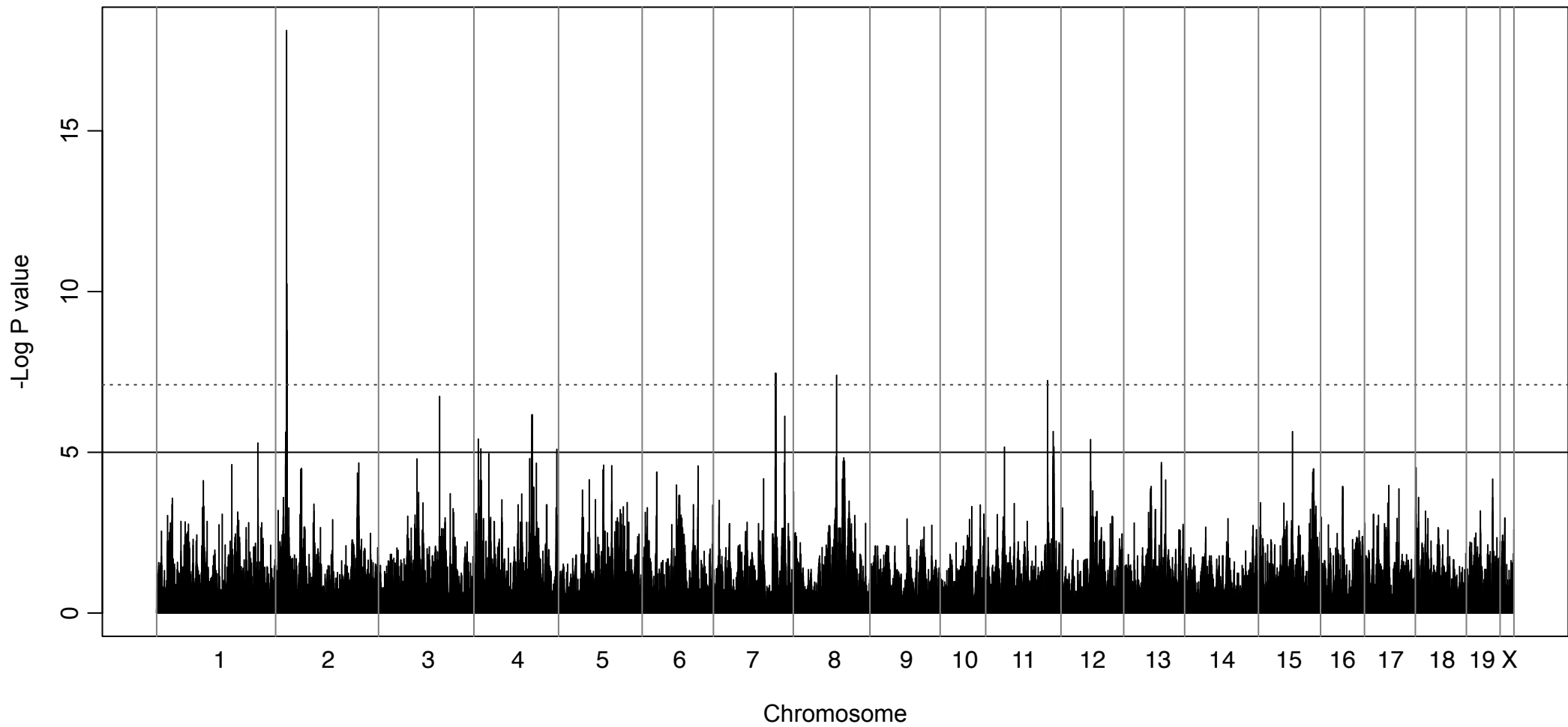

Supplement: Figure S1 — Genome-wide association mapping without discordant AKR/J and SM/J strains. EMMA analysis was conducted as described earlier (see Materials and Methods, and Figure 2) while omitting the AKR/J and SM/J discordant strains. -Log10P values are depicted and represent genome wide significance of association for each SNP. Standardized threshold was set at P value 1.0×10^−5^ (solid line) and the Bonferroni multiple testing correction threshold was calculated to be 7.87×10^−8^ (dashed line). (PDF) [file pone.0018957.s001.pdf]
