# Supplementary material for: Genetic Control of Susceptibility to Infection with Candida albicans in Mice
Source: PLoS One. 2011 Apr 20;6(4):e18957. doi: 10.1371/journal.pone.0018957 (PMC3080400; doi:10.1371/journal.pone.0018957)
Supplement: Table S1 — In silico identified loci controlling response to C. albicans infection in inbred mouse strains. SNPs that have passed the Bonferroni cutoff (α = 0.01, P value = 7.99×10^−8^) are shaded in gray. (PDF) [file pone.0018957.s002.pdf]

| Chromosome | Position (Mb) | P value | $-Log_{10}$ P value |
|---|---|---|---|
| 1 | 61.10 | 4.96E-06 | 5.30 |
| 2 | 31.14 | 6.66E-06 | 5.18 |
| | 31.19 | 6.66E-06 | 5.18 |
| | 31.22 | 2.30E-06 | 5.64 |
| | 31.29 | 6.66E-06 | 5.18 |
| | 33.74 | 6.51E-06 | 5.19 |
| | 33.74 | 6.70E-07 | 6.17 |
| | 33.74 | 3.04E-10 | 9.52 |
| | 33.76 | 8.71E-07 | 6.06 |
| | 33.82 | 2.43E-11 | 10.61 |
| | 33.90 | 8.71E-07 | 6.06 |
| | 33.92 | 5.74E-06 | 5.24 |
| | 33.95 | 1.36E-06 | 5.87 |
| | 33.96 | 4.65E-09 | 8.33 |
| | 34.45 | 2.28E-06 | 5.64 |
| | 34.75 | 7.78E-06 | 5.11 |
| 4 | 106.21 | 2.81E-06 | 5.55 |
| | 111.05 | 7.69E-06 | 5.11 |
| | 111.17 | 7.69E-06 | 5.11 |
| | 111.20 | 7.69E-06 | 5.11 |
| | 111.24 | 7.69E-06 | 5.11 |
| | 136.62 | 1.17E-07 | 6.93 |
| 6 | 120.43 | 2.32E-06 | 5.63 |
| 7 | 122.15 | 2.33E-06 | 5.63 |
| | 133.22 | 1.31E-07 | 6.88 |
| 8 | 78.25 | 7.43E-07 | 6.13 |
| | 87.98 | 3.43E-06 | 5.46 |
| | 88.00 | 3.43E-06 | 5.46 |
| | 88.00 | 3.43E-06 | 5.46 |
| | 88.02 | 3.43E-06 | 5.46 |
| | 88.24 | 5.77E-06 | 5.24 |
| | 88.38 | 3.43E-06 | 5.46 |
| | 88.39 | 2.26E-06 | 5.65 |
| | 88.63 | 3.43E-06 | 5.46 |
| | 89.49 | 3.43E-06 | 5.46 |
| 11 | 32.27 | 4.01E-06 | 5.40 |
| | 98.87 | 7.63E-09 | 8.12 |
| | 98.91 | 7.17E-06 | 5.14 |
| | 98.91 | 7.17E-06 | 5.14 |
| | 108.16 | 6.66E-07 | 6.18 |
| | 109.16 | 3.25E-06 | 5.49 |
| | 109.17 | 3.25E-06 | 5.49 |
| | 109.17 | 8.39E-06 | 5.08 |
| 12 | 50.93 | 9.02E-07 | 6.04 |
| 15 | 54.18 | 5.19E-06 | 5.28 |
