# Supplementary material for: Genetic Control of Susceptibility to Infection with Candida albicans in Mice
Source: PLoS One. 2011 Apr 20;6(4):e18957. doi: 10.1371/journal.pone.0018957 (PMC3080400; doi:10.1371/journal.pone.0018957)
Supplement: Table S2 — Candidate genes in the Carg3 region. A total of 59 genes containing IFNγ-inducible STAT1 binding sites and their overall mRNA expression (>2X) upon IFNγ stimulation in Hela cells are represented. Genes considered for further prioritization had a high (>20) ChIP-Seq peak height and a significant (>2X) gene expression. N/A designation in the gene expression column was given for genes that were not represented on the microarray. (PDF) [file pone.0018957.s003.pdf]

Table S2 (Radovanovic *et al.*)

Genes in *Carg3* region containing IFNg-inducible STAT1 binding sites and their overall mRNA expression (>2X) upon IFNg stimulation i

59 genes

Gene symbol	Gene name	ChIP-Seq Peak height ≥ 20	Gene expression Orange=Up, Green=Down
2310036O22Rik	RIKEN cDNA 2310036O22 gene		N/A
4921524J17Rik	RIKEN cDNA 4921524J17 gene		N/A
Abcc12	ATP-binding cassette, sub-family C (CFTR/MRP), member 12		
Adcy7	adenylate cyclase 7	✓	Orange
Asf1b	ASF1 anti-silencing function 1 homolog B (S. cerevisiae)	✓	
Best2	bestrophin 2	✓	
Cacna1a	calcium channel, voltage-dependent, P/Q type, alpha 1A subunit	✓	
Calr	calreticulin		Orange
Ccdc130	coiled-coil domain containing 130		
Cd97	CD97 antigen		N/A
Chd9	chromodomain helicase DNA binding protein 9		Orange
Dand5	DAN domain family, member 5		Green
Dcaf15	DDB1 and CUL4 associated factor 15		N/A
Dhps	deoxyhypusine synthase		
Dnaja2	DnaJ (Hsp40) homolog, subfamily A, member 2	✓	Orange
Dnajb1	DnaJ (Hsp40) homolog, subfamily B, member 1	✓	
Elmod2	ELMO domain containing 2		
Farsa	phenylalanyl-tRNA synthetase, alpha subunit		N/A
Gab1	growth factor receptor bound protein 2-associated protein 1	✓	Orange
Gadd45gip1	growth arrest and DNA-damage-inducible, gamma interacting protein 1	✓	
Gcdh	glutaryl-Coenzyme A dehydrogenase		Orange
Gipc1	GIPC PDZ domain containing family, member 1	✓	
Gm16994	predicted gene, 16994		N/A
Gpt2	glutamic pyruvate transaminase (alanine aminotransferase) 2	✓	
Hook2	hook homolog 2 (Drosophila)	✓	
Ier2	immediate early response 2		
Il15	interleukin 15	✓	
Inpp4b	inositol polyphosphate-4-phosphatase, type II	✓	Orange
Itfg1	integrin alpha FG-GAP repeat containing 1	✓	
Junb	Jun-B oncogene	✓	
Lonp2	lon peptidase 2, peroxisomal		
Mir1199	microRNA 1199		N/A
Mir181c	microRNA 181c		N/A
Mir181d	microRNA 181d		N/A
Mir23a	microRNA 23a	✓	N/A
Mir24-2	microRNA 24-2	✓	N/A
Mir27a	microRNA 27a	✓	N/A
N4bp1	NEDD4 binding protein 1		
Nacc1	nucleus accumbens associated 1, BEN and BTB (POZ) domain containing		
Nanos3	nanos homolog 3 (Drosophila)		
Ndufb7	NADH dehydrogenase (ubiquinone) 1 beta subcomplex, 7		
Nfix	nuclear factor I/X		
Papd5	PAP associated domain containing 5		Orange
Phkb	phosphorylase kinase beta		
Pkn1	protein kinase N1		
Prdx2	peroxiredoxin 2		
Prkaca	protein kinase, cAMP dependent, catalytic, alpha		Orange
Rnaseh2a	ribonuclease H2, large subunit		
Rnf150	ring finger protein 150		Green
Rtbdn	retbindin		
Samd1	sterile alpha motif domain containing 1	✓	
Scoc	short coiled-coil protein		
Siah1a	seven in absentia 1A	✓	N/A
Tbc1d9	TBC1 domain family, member 9		Green
Tecr	trans-2,3-enoyl-CoA reductase		N/A
Tmem188	transmembrane protein 188	✓	
Trmt1	TRM1 tRNA methyltransferase 1 homolog (S. cerevisiae)		Orange
Vps35	vacuolar protein sorting 35		Orange
Zfp330	zinc finger protein 330	✓	N/A
