# Supplementary material for: Genetic Control of Susceptibility to Infection with Candida albicans in Mice
Source: PLoS One. 2011 Apr 20;6(4):e18957. doi: 10.1371/journal.pone.0018957 (PMC3080400; doi:10.1371/journal.pone.0018957)
Supplement: Table S3 — Candidate genes in the Carg4 region. A total of 147 genes containing IFNγ-inducible STAT1 binding sites and their overall mRNA expression (>2X) upon IFNγ stimulation in Hela cells are represented. Genes considered for further prioritization had both high (>20) ChIP-Seq peak height and a significant (>2X) gene expression. N/A designation in the gene expression column was given for genes that were not represented on the microarray. (PDF) [file pone.0018957.s004.pdf]

Table S3 (Radovanovic *et al.*)

Genes in *Carg4* region containing IFNg-inducible STAT1 binding sites and their overall mRNA expression (>2X) upon IFNg stimulation in Hela cells

147 genes

Gene symbol	Gene name	ChIP-Seq Peak height ≥ 20	Gene expression Orange=Up, Green=Down
1110035M17Rik	RIKEN cDNA 1110035M17 gene		N/A
1700028N14Rik	RIKEN cDNA 1700028N14 gene		N/A
1810046J19Rik	RIKEN cDNA 1810046J19 gene		N/A
Aarsd1	alanyl-tRNA synthetase domain containing 1		
Abcc3	ATP-binding cassette, sub-family C (CFTR/MRP), member 3	✓	
Acly	ATP citrate lyase		
Acsf2	acyl-CoA synthetase family member 2		
Ankrd40	ankyrin repeat domain 40	✓	
Arhgap23	Rho GTPase activating protein 23		
Arl4d	ADP-ribosylation factor-like 4D		
Asb16	ankyrin repeat and SOCS box-containing 16	✓	
Atp6v0a1	ATPase, H+ transporting, lysosomal V0 subunit A1	✓	
Atxn7l3	ataxin 7-like 3		
BC030867	cDNA sequence BC030867	✓	N/A
Becn1	beclin 1, autophagy related	✓	
Brca1	breast cancer 1		
Cacna1g	calcium channel, voltage-dependent, T type, alpha 1G subunit		
Cacnb1	calcium channel, voltage-dependent, beta 1 subunit		
Calcoco2	calcium binding and coiled-coil domain 2		
Ccdc103	coiled-coil domain containing 103		
Ccdc43	coiled-coil domain containing 43	✓	
Ccr10	chemokine (C-C motif) receptor 10		
Ccr7	chemokine (C-C motif) receptor 7	✓	
Cd300lg	CD300 antigen like family member G		
Cdc6	cell division cycle 6 homolog (S. cerevisiae)	✓	
Cdk12	cyclin-dependent kinase 12		N/A
Cnp	2',3'-cyclic nucleotide 3' phosphodiesterase		
Coasy	Coenzyme A synthase	✓	
Col1a1	collagen, type I, alpha 1		
Cwc25	CWC25 spliceosome-associated protein homolog (S. cerevisiae)		N/A
Dcakd	dephospho-CoA kinase domain containing		
Dhx8	DEAH (Asp-Glu-Ala-His) box polypeptide 8	✓	
Dlx4	distal-less homeobox 4		
Dnajc7	DnaJ (Hsp40) homolog, subfamily C, member 7		
E130012A19Rik	RIKEN cDNA E130012A19 gene		N/A
Eftud2	elongation factor Tu GTP binding domain containing 2		
Eif1	eukaryotic translation initiation factor 1		
Epn3	epsin 3		
Fam117a	family with sequence similarity 117, memberA	✓	
Fmnl1	formin-like 1		
Fmnl1	formin-like 1		
G6pc3	glucose 6 phosphatase, catalytic, 3		
Gfap	glial fibrillary acidic protein		
Ghdc	GH3 domain containing		
Gjd3	gap junction protein, delta 3		
Gosr2	golgi SNAP receptor complex member 2	✓	
Gpatch8	G patch domain containing 8		
Grb7	growth factor receptor bound protein 7		
Grn	granulin		
Hap1	huntingtin-associated protein 1	✓	
Hdac5	histone deacetylase 5		
Hexim1	hexamethylene bis-acetamide inducible 1		
Hexim2	hexamthylene bis-acetamide inducible 2	✓	
Hsd17b1	hydroxysteroid (17-beta) dehydrogenase 1		
Ifi35	interferon-induced protein 35	✓	
Igf2bp1	insulin-like growth factor 2 mRNA binding protein 1	✓	
Igfbp4	insulin-like growth factor binding protein 4		
Ikzf3	IKAROS family zinc finger 3	✓	
Itga3	integrin alpha 3	✓	
Jup	junction plakoglobin		
Kif18b	kinesin family member 18B		
Krt10	keratin 10		
Krt15	keratin 15		
Krt16	keratin 16	✓	
Krt17	keratin 17	✓	
Krt24	keratin 24	✓	
Krt32	keratin 32		
Krt42	keratin 42		N/A
Lasp1	LIM and SH3 protein 1	✓	N/A
Lrrc46	leucine rich repeat containing 46		
Lrrc59	leucine rich repeat containing 59		
Lsm12	LSM12 homolog (S. cerevisiae)		
Luc7l3	LUC7-like 3 (S. cerevisiae)	✓	N/A
Lyzl6	lysozyme-like 6		
Map3k14	mitogen-activated protein kinase kinase kinase 14		

Med1	mediator complex subunit 1		
Mllt6	myeloid/lymphoid or mixed-lineage leukemia (trithorax homolog, Drosophila); translocated to, 6	✓	
Mpp3	membrane protein, palmitoylated 3 (MAGUK p55 subfamily member 3)	✓	
Mrpl10	mitochondrial ribosomal protein L10		
Mrpl45	mitochondrial ribosomal protein L45	✓	
Msl1	male-specific lethal 1 homolog (Drosophila)		
Myst2	MYST histone acetyltransferase 2	✓	
Naglu	alpha-N-acetylglucosaminidase (Sanfilippo disease IIIB)	✓	
Nags	N-acetylglutamate synthase	✓	
Nbr1	neighbor of Brca1 gene 1		
Nbr1	neighbor of Brca1 gene 1		
Nfe2l1	nuclear factor, erythroid derived 2,-like 1		
Ngfr	nerve growth factor receptor (TNFR superfamily, member 16)		
Nkiras2	NFKB inhibitor interacting Ras-like protein 2		
Nme1	non-metastatic cells 1, protein (NM23A) expressed in	✓	
Nme2	non-metastatic cells 2, protein (NM23B) expressed in		N/A
Nmt1	N-myristoyltransferase 1	✓	
Nr1d1	nuclear receptor subfamily 1, group D, member 1	✓	
Nxph3	neurexophilin 3		
Osbpl7	oxysterol binding protein-like 7		
Pcgf2	polycomb group ring finger 2	✓	
Pgap3	post-GPI attachment to proteins 3		N/A
Plcd3	phospholipase C, delta 3		
Plekhh3	pleckstrin homology domain containing, family H (with MyTH4 domain) member 3		
Plxdc1	plexin domain containing 1	✓	
Pnpo	pyridoxine 5'-phosphate oxidase		
Ppp1r9b	protein phosphatase 1, regulatory subunit 9B	✓	
Ppy	pancreatic polypeptide		
Psmb3	proteasome (prosome, macropain) subunit, beta type 3	✓	
Psmd3	proteasome (prosome, macropain) 26S subunit, non-ATPase, 3	✓	
Ptrf	polymerase I and transcript release factor	✓	
Rab5c	RAB5C, member RAS oncogene family	✓	
Rapgefl1	Rap guanine nucleotide exchange factor (GEF)-like 1		
Rara	retinoic acid receptor, alpha	✓	
Rdm1	RAD52 motif 1		
Rpl19	ribosomal protein L19	✓	
Rpl27	ribosomal protein L27	✓	
Rsad1	radical S-adenosyl methionine domain containing 1		
Rundc1	RUN domain containing 1	✓	
Samd14	sterile alpha motif domain containing 14		
Sgca	sarcoglycan, alpha (dystrophin-associated glycoprotein)		
Sh3d20	SH3 domain containing 20		
Skap1	src family associated phosphoprotein 1		
Slc35b1	solute carrier family 35, member B1		
Slc4a1	solute carrier family 4 (anion exchanger), member 1		N/A
Snf8	SNF8, ESCRT-II complex subunit, homolog (S. cerevisiae)		
Snx11	sorting nexin 11		
Sost	sclerostin		
Spop	speckle-type POZ protein		
Srcin1	SRC kinase signaling inhibitor 1	✓	N/A
Stac2	SH3 and cysteine rich domain 2		
Stard3	START domain containing 3	✓	
Stat3	signal transducer and activator of transcription 3	✓	
Stat5a	signal transducer and activator of transcription 5A	✓	
Stat5b	signal transducer and activator of transcription 5B	✓	
Thra	thyroid hormone receptor alpha		
Tmub2	transmembrane and ubiquitin-like domain containing 2	✓	
Tns4	tensin 4	✓	
Tob1	transducer of ErbB-2.1	✓	
Top2a	topoisomerase (DNA) II alpha		
Ttc25	tetratricopeptide repeat domain 25		
Ttll6	tubulin tyrosine ligase-like family, member 6		
Tubg1	tubulin, gamma 1		
Tubg2	tubulin, gamma 2	✓	
Ube2z	ubiquitin-conjugating enzyme E2Z (putative)		
Ubtf	upstream binding transcription factor, RNA polymerase I	✓	
Vps25	vacuolar protein sorting 25 (yeast)		
Wfikkn2	WAP, follistatin/kazal, immunoglobulin, kunitz and netrin domain containing 2		
Wipf2	WAS/WASL interacting protein family, member 2		
Wnt3	wingless-related MMTV integration site 3	✓	
Xylt2	xylosyltransferase II		
Zfp652	zinc finger protein 652		N/A
